# Gene ontology analysis of transcriptome data from DMBA-induced mammary tumors of rats fed a high-corn oil and a high-extra virgin olive oil diet

**DOI:** 10.1016/j.dib.2018.11.135

**Published:** 2018-12-07

**Authors:** Raquel Escrich, Marta Cubedo, Eduard Escrich, Raquel Moral

**Affiliations:** a Department of Cell Biology, Physiology and Immunology, Physiology Unit, Faculty of Medicine, Universitat Autònoma de Barcelona, 08193 Bellaterra, Barcelona, Spain; b Department of Statistics, Universitat de Barcelona, 08028 Barcelona, Spain

## Abstract

Breast cancer is the most common malignancy in women worldwide, and dietary lipids are important environmental factors influencing its etiology. In this work we present data in relation to the transcriptional effects of two high-fat diets, one high in corn oil (HCO) and one high in extra-virgin olive oil (HOO), administered from weaning or after induction, on 7,12-dimethylbenz(a)anthracene (DMBA)-induced rat mammary tumors. Raw data were deposited at ArrayExpress under accession number E-MTAB-3541. We compared the gene expression profiles of the mammary tumors from the high-fat diet groups with those from the control group, finding different effects of diets depending on timing and type of dietary intervention. Lists of differentially expressed genes were analyzed to find overrepresented categories of biological significance. Here we provide information about the cell functions categories overrepresented in significantly modulated genes by effect of the high-fat diets. Further investigations of such functions are described in “A high corn oil diet strongly stimulates mammary carcinogenesis, while a high extra virgin olive oil diet has a weak effect, through changes in metabolism, immune system function, and proliferation/apoptosis pathways” (Escrich et al., in press) [1].

## Specifications table

**Table:** 

Subject area	Biology
More specific subject area	Nutrition and cancer
Type of data	Tables (online), figure
How data was acquired	Microarray Analysis Suite 5.0 (Affymetrix GeneChip^®^)
Data format	Analyzed
Experimental factors	DMBA-induced mammary adenocarcinomas from rats fed a low-fat diet, a high-corn oil diet or a high-extra virgin olive oil diet
Experimental features	Female Sprague-Dawley rats fed with low-fat, a high-corn oil diet or a high-extra virgin olive oil diet from weaning or after induction with DMBA at 53 days of age. Total RNA was isolated from mammary adenocarcinomas of each condition for whole-genome gene expression profiling
Data source location	Universitat Autònoma de Barcelona, Cerdanyola del Vallès, Barcelona, Spain
Data accessibility	Data is with this article and raw microarray is available at ArrayExpress accession number E-MTAB-3541, https://www.ebi.ac.uk/arrayexpress/experiments/E-MTAB-3541/
Related research article	Escrich R, Costa I, Moreno M, Cubedo M, Vela E, Escrich E, Moral R. A high corn oil diet strongly stimulates mammary carcinogenesis, while a high extra virgin olive oil diet has a weak effect, through changes in metabolism, immune system function, and proliferation/apoptosis pathways. J Nutr Biochem, in press [1]

## Value of the data

• This data shows different effects of a high-corn oil and a high-extra virgin olive oil diet and is valuable for researchers interested in the effects of dietary compounds on gene expression profile.
• This data is valuable for researchers interested in the effects of dietary lipids on breast cancer.
• Gene ontology analysis identifies cell functions that may be useful in investigations related to the different biological effects of high-fat diets.

## 1 Data

**Fig. 1:**
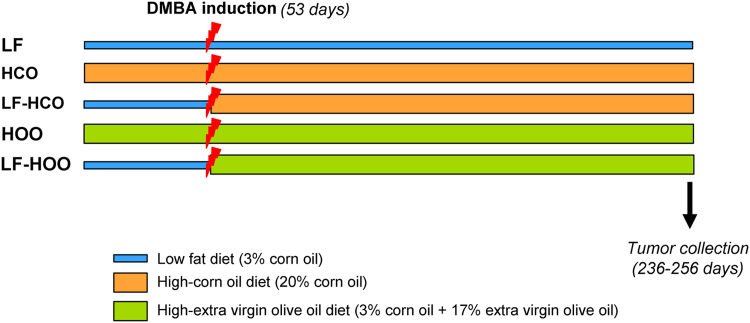
Experimental design. Female rats were fed the control low-fat diet (LF), the high-corn oil diet from weaning (HCO) or from induction (LF-HCO), and the high olive oil diet from weaning (HOO) or from induction (LF-HOO). Animals were induced with 5 mg of dimethylbenz[α]anthracene (DMBA) at 53 days of age and euthanized at 236–256 days.

We characterized the transcriptional effects of two high-fat diets, in comparison to a control low-fat diet, on experimental 7,12-dimethylbenz(a)anthracene (DMBA)-induced mammary tumors. Female Sprague-Dawley rats were fed with a control diet, or with a high-corn oil or a high extra virgin olive oil from weaning or after induction. RNA was isolated to assess the gene expression profile of mammary tumors at 246 days of age. Identification of genes down- or up-regulated by the high-fat diets was obtained and gene ontology analyses of differentially expressed genes using FatiGo and Genecodis from Babelomic platform were performed. Data presented include gene ontology of all significantly modulated genes in the mammary tumors from each high-fat diet group, and analysis of genes specifically modulated in some groups (not overlapped in common between high-fat diet groups). Further investigations of the functions modulated by the high-fat diets on experimental breast cancer are described in [1]. Data is available online at https://www.ebi.ac.uk/arrayexpress/experiments/E-MTAB-3541/, and Fig. 1. with this article.

## 2 Experimental design, materials, and methods

### 2.1 Animals and experimental design

Female Sprague-Dawley Crl:SD rats were obtained at 23 days of age from Charles River Lab (L’Arbresle Cedex, France), housed 2–3 per cage and maintained in a controlled environment of 12 h light:12 h dark cycle. Animals were distributed depending to the type and timing of dietary intervention (*n* = 20 each group). The LF control group was fed a 3% corn oil (w/w) low-fat diet from weaning. Animals from the HCO group were fed a high-corn oil diet (20% corn oil -w/w-) from weaning, while the LF-HCO group was fed the control diet until induction and then the HCO diet until the end of the experiment. Animals from HOO were fed a high-olive oil diet (3% con oil + 17% extra virgin olive oil -w/w-) from weaning, while the LF-HOO group was fed the control diet until induction and then changed to the HOO diet. The composition, preparation and suitability of the experimental diets used have been previously described [2], [3], [4]. At 53 days of age mammary cancer was induced by intragastric gavage with one single dose of 5 mg of DMBA (Sigma-Aldrich, St. Louis, MO) dissolved in 1 mL of corn oil. At the end of the assay rats were euthanized by decapitation on days 236–256. Tumors were flash-frozen and stored at -80 °C. Only mammary adenocarcinomas confirmed by histopathological examination have been included in this study (Fig. 1).

### 2.2 RNA extraction and microarrays

Total RNA from mammary tumors was isolated using the RNeasy^®^ Extraction Kit (QiaGen, Hilden, Germany). RNA was quantified with Nanodrop 1000 (ThermoFisher Scientific Inc, Waltham, MA, USA), while quality and integrity was analyzed by Agilent Bioanalyzer 2100 (Agilent Technologies, Santa Clara, CA, USA) and by ethidium bromide-stained agarose gel electrophoresis. For each group, six mammary adenocarcinomas were chosen for whole-genome gene expression profiling with GeneChip^®^ Rat Exon 1.0 ST Array filters containing probesets for 850,000 exons (Affymetrix, Santa Clara, CA, USA). Three μg of high quality RNA (integrity number > 8) were labeled with One-Cycle Target Labeling and Control Reagent (Affymetrix). Labeled samples were then hybridized to chips using the hybridization oven 640, (Affymetrix), and scanned with GeneChip Scanner 3000 7G (Affymetrix) in the Microarrays Service from Vall d’Hebron Research Institute (VHIR).

### 2.3 Data analysis

The scanned images obtained were processed with Microarray Analysis Suite 5.0 (Affymetrix). Raw expression values were first preprocessed using the RMA method [5], and data was then non-specific filtered to remove low signal and low variability genes. Microarray data have been deposited at ArrayExpress and is available under accession number E-MTAB-3541.

Statistical analysis of data was carried out using language R (www.bioconductor.org), and selection of genes differentially expressed between conditions was based on a linear model with empirical Bayes moderation [6]. Genes were classified into ranks according to its differential expression. To control of the false discovery rate in multiple testing, p-values were adjusted using the Benjamini and Hochberg method [7].

We obtained the lists of differentially expressed genes in the high-fat diet groups in comparison to the control low-fat diet group. Analyses of Biological Significance were based on Overrepresentation (enrichment) tests which establish if the genes that have been found as differentially expressed are present more than would be expected (over-represented) in specific Gene Ontology (GO) categories. Gene ontology analyses were carried out comparing the lists of genes against the rest of the genome. Enrichment tests were performed with FatiGO and Genecodis from Babelomics tool, applying Fisher׳s exact test for 2 × 2 contingency tables and multiple test correction to find significant over-represented GO terms. Functional analysis with FatiGO is presented in Supplementary Excel file. Supplementary tables present the analysis with Genecodis, setting GO annotations at level 3–6. Supplementary Table 1 present the analysis of all the genes differentially modulated in each high-fat diet group. We also performed the analysis dividing the clusters of genes in commonly modulated and specific for each group. Only genes specific for LF-HCO and HOO groups showed significantly enriched categories (Supplementary Table 2).

## Appendix A Supplementary material

Supplementary material: Functional analysis with Genecodis: Enriched Gene Ontology (GO) categories in genes down- and up-modulated in the high-fat diets groups.

Supplementary material: Functional analysis with Genecodis: Enriched Gene Ontology (GO) categories in genes only modulated in specific high-fat diets groups.

Supplementary material: Functional analysis with FatiGo: Enriched Gene Ontology (GO) categories in genes down- and up-regulated in the high-fat diets groups.
